# Slope Entropy Characterisation: An Asymmetric Approach to Threshold Parameters Role Analysis

**DOI:** 10.3390/e26010082

**Published:** 2024-01-18

**Authors:** Mahdy Kouka, David Cuesta-Frau, Vicent Moltó-Gallego

**Affiliations:** 1 Department of System Informatics and Computers, Universitat Politècnica de València, 03801 Alcoy, Spain; mkouka@doctor.upv.es (M.K.); vimolgal@epsa.upv.es (V.M.-G.); 2 Technological Institute of Informatics, Universitat Politècnica de València, 03801 Alcoy, Spain

**Keywords:** Slope Entropy, time series classification, parameter optimisation

## Abstract

Slope Entropy (SlpEn) is a novel method recently proposed in the field of time series entropy estimation. In addition to the well-known embedded dimension parameter, *m*, used in other methods, it applies two additional thresholds, denoted as δ and γ, to derive a symbolic representation of a data subsequence. The original paper introducing SlpEn provided some guidelines for recommended specific values of these two parameters, which have been successfully followed in subsequent studies. However, a deeper understanding of the role of these thresholds is necessary to explore the potential for further SlpEn optimisations. Some works have already addressed the role of δ, but in this paper, we extend this investigation to include the role of γ and explore the impact of using an asymmetric scheme to select threshold values. We conduct a comparative analysis between the standard SlpEn method as initially proposed and an optimised version obtained through a grid search to maximise signal classification performance based on SlpEn. The results confirm that the optimised version achieves higher time series classification accuracy, albeit at the cost of significantly increased computational complexity.

## 1. Introduction

Time series entropy analysis plays a fundamental role in various scientific domains, ranging from biology and medicine to finance and many engineering fields [1,2,3,4,5,6]. It provides valuable insights into the complexity and predictability of such time series, and can be used as a distinguishing feature for signal classification [7,8,9,10].

For example, Permutation Entropy (PE) [11], a popular method in this time series analysis framework, provides a means to quantify the disorder or randomness in a time series by examining the order of values within the series. It has found applications in those diverse domains stated before and is valuable for characterising complex systems [12,13]. Specifically, it has been successfully used in medicine [14,15,16,17], engineering [3,18,19,20], economics [21,22,23], and natural sciences [5,24,25], to cite just a few examples.

Sample Entropy (SampEn) is another widely used technique for assessing the regularity and self-similarity of time series data. It measures the similarity of subsequences within the time series, making it a useful tool for detecting complex patterns and anomalies in various applications. In the domain of healthcare and biomedical research, SampEn aids in the assessment of heart rate variability [26], electroencephalography (EEG) data [27], and, in general, the detection of pathological conditions, contributing to improved patient diagnostics and monitoring. In finance, SampEn proves valuable in modelling and forecasting financial time series, helping analysts make informed decisions in an ever-changing market landscape [28]. Furthermore, in the realm of environmental science, SampEn can also support the identification of complex patterns in climatic data, facilitating better understanding and management of the underlying interacting processes [29].

Among these and many other entropy estimation methods available nowadays, Slope Entropy (SlpEn) has emerged as a novel approach that shows promise in capturing the intricate patterns of time series data for such classification purposes. SlpEn, introduced in the work by [30], incorporates two additional input parameters, thresholds δ and γ, to derive a symbolic representation of data subsequences according to the differences between consecutive time series samples, as will be described in detail later in Section 2.2. This characteristic of SlpEn allows for a more refined and customised analysis of time series patterns, although it also entails more input parameters than other methods, such as PE [11,31].

The original SlpEn paper provided initial guidelines for selecting specific values of δ and γ and facilitating adoption, guidelines which have since been successfully applied in subsequent SlpEn studies [32,33]. A few other works have already explored the influence of the δ parameter value on SlpEn classification potential [34], but a thorough investigation into the roles of both δ and γ is still essential to fully exploit SlpEn’s capabilities. This is the same kind of performance characterisation analysis that has been performed for other entropy methods in the past.

In order to illustrate this point, let us consider again methods PE and SampEn. On one hand, in the case of PE, parameter influence or selection has also been the focus of many studies, like [31,35]. Algorithm speed is also crucial in many applications, and characterisation and improvements in this regard are also frequent, like the study [36]. On the other hand, in the case of SampEn, there are studies to optimise input parameter threshold *r* to improve the detection of cardiovascular diseases using RR interval time series [37]. Similarly, the work [38] describes how to optimise all SampEn parameters *N*, *m*, and *r*, for the prediction of paroxysmal atrial fibrillation (AF) termination and the electrical cardioversion outcome in persistent AF. In terms of computational cost optimisations, there are also studies like [39]. There are even specific studies to optimise the implementation of SampEn in wearable devices [27].

In this paper, we aim to expand the knowledge of SlpEn by conducting a more ambitious analysis of its δ and γ parameters’ influence on time series classification performance and exploring the possible benefits of employing an asymmetric scheme to choose the threshold values. In other words, employing different threshold values for positive and negative differences between consecutive samples. This will certainly double the number of thresholds, but we hypothesise that this way, it will be possible to improve the matching between SlpEn capabilities and features of the time series under analysis.

We compared the classification accuracy achieved, defined later, using the standard SlpEn method [30] with that from an improved version obtained by applying the asymmetric scheme mentioned above. Both methods used a grid parameter search to maximise signal classification accuracy instead of just the recommended input values. To evaluate the effectiveness of the optimised version, we considered publicly available benchmark datasets from different domains, as described in Section 2.1.

The expected contributions of this paper are twofold: Firstly, we shed light on the roles of δ and γ in the SlpEn method, providing more insights into their significance and potential for fine-tuning in difficult signal classification problems. Secondly, we demonstrated that the optimised version of SlpEn achieves higher classification accuracy, although at a significantly increased computational cost that will not probably pay-off in all cases. This trade-off between accuracy and computational complexity will have to be balanced, allowing researchers to make informed decisions based on specific application requirements.

The rest of the paper is organised as follows: Section 2, Materials and Methods, presents a comprehensive review of the specific time series datasets used in the experiments, addressing different application fields. This section also contains a detailed description of the SlpEn method in its original version and the variations that are applied in the present study, including the experimental setup and the grid search procedure for optimising SlpEn. In Section 3, we present the results of the comparative analysis in terms of signal classification accuracy achieved in each case. The discussion takes place in Section 4, where we evaluate the implications of employing an asymmetric scheme for threshold selection. Finally, Section 5 concludes the paper and highlights potential future research directions to further enhance SlpEn’s applicability and performance in diverse scientific contexts.

## 2. Materials and Methods

This section presents first the time series utilised in the experiments. All of these datasets are publicly available and have been extensively employed in numerous similar studies. They hold a well-established reputation as representative time series for analysis, facilitating direct result comparison with previous research. Furthermore, the dataset selection is characterised by its diversity and variation, mitigating potential interpretation biases and ensuring a higher potential for generalisation.

Once the experimental datasets have been introduced, this section also describes in detail the method under analysis, Slope Entropy. Specifically, we introduce the standard method based on input parameters *m*, δ, and γ, and then we describe the modifications proposed to use an extended asymmetrical version of the two thresholds. These two versions will be the objective of the comparative classification accuracy performance analysis carried out in the experiments section (Section 3).

### 2.1. Datasets

As stated above, in order to assess the efficacy and resilience of the proposed SlpEn improvements, it is imperative to conduct experiments on diverse datasets exhibiting variations in length, level of ties, and regularity, among other time series properties. Thus, the specific datasets utilised in the present study are:

- The Bern–Barcelona EEG database [40]. This database comprises a collection of both focal and non-focal time series extracted from seizure-free recordings of patients afflicted with pharmacoresistant focal-onset epilepsy. For the purpose of our experiments, we employed 50 records, each possessing a length of 10,240 data points, sampled at a frequency of 512 Hz. This database has also been used in works such as [41], which includes a review of results achieved in other classification studies based on time series from this same database.
- The Fantasia RR database [42]: It presents a meticulously curated repository comprising a total of 40 individual time series, thoughtfully stratified into two cohorts of 20 records each (mature subjects and a counterpart assembly of youthful subjects). All subjects were, in principle, healthy, thereby obviating possible confounding health-related factors. They were monitored over an extended period of 120 min, with a sampling frequency of 250 Hz. This database has been used in many studies, such as [43,44].
- The Ford A dataset [45]: It is a repository of data gleaned from an automotive subsystem. The principal objective underpinning its creation was the empirical evaluation of the efficacy of classification schemes upon the acoustic characteristics of engine noise. Within the ambit of this experimental undertaking, a corpus of 40 discrete records was selected and subsequently employed for analysis from each distinct class.
- The House Twenty dataset [46,47]: It is a compendium of temporal sequences emanating from 40 distinct domestic entities. They are part of the Personalised Retrofit Decision Support Tools for UK Homes using Smart Home Technology (REFIT) project. This dataset includes data from 40 households, divided into two classes with 20 each: The first class represents the consumption of electricity in general, and the second class represents the specific electrical consumption of dryers and washing machines. We also used this dataset in our previous work [34].
- The PAF (Paroxysmal Atrial Fibrillation) prediction dataset [48]: This dataset comprises discrete 5-min temporal recordings corresponding to patients diagnosed with PAF. These temporal records were classified into two distinct categories: The first pertains to recordings that immediately precede the onset of a PAF episode, while the second encompasses instances temporally distant from any PAF manifestation. Each classification category comprises a total of 25 distinct files. This is a very well-known dataset used in a myriad of scientific works [49,50,51].
- The Worms two-class dataset [52,53]: It contains a time series intrinsically linked to the locomotive patterns exhibited by a distinct species of worm used in the realm of behavioural genetics research. We selected records from two classes: mutant and non-mutant worms. The first type contains 75 records of 900 samples, and the second type has 105 records with the same time series length. As with previous datasets, there are other works that used time series from this one [54,55].
- The Bonn EEG dataset [56,57]. This dataset encapsulates a corpus of 4097 electroencephalograms, each one with a duration of 23.6 s. These instances are distinctly categorised into five salient classes (A, B, C, D, and E), reflecting the underlying diversity of neural activity scenarios under consideration. Specifically, the classes include healthy subjects with eyes open (Class A) and those with eyes closed (Class B). Other instances pertain to epileptic subjects classified as Class C, D, and E (see further details in [57]). For the scope of the specific experiments in the present paper, the focus was directed solely towards classes D and E (seizure-free periods at the epileptogenic zone, and seizure activity from the hippocampal focus), with 100 records from each class. There are many examples available of works using this same dataset [58,59,60].
- The Synthetic database. As its name suggests, it is a collection of datasets that have been generated artificially by a computer. It is composed of three different sets: Synth1, Synth2, and Synth3. Each one of them is composed of two classes; the first one is generated by a normal (Gaussian) distribution, while the other is based on a uniform distribution. Each class contains 20 time series with a length of 3000 samples. Regarding the parameters used to generate the series, Synth1 uses mean = 0 and standard deviation equal to 5 (SD = 5) for its Gaussian class. Synth2’s Gaussian class uses mean = 0 and SD = 10. In the case of Synth3, mean = 0 and SD = 20. On the other hand, the uniform distribution used to generate the second class uses the same parameters for all three datasets, drawing samples uniformly from the range [−1, 1]. It has been included for reference purposes, but it is not used in all the experiments since it is not as illustrative as the real datasets.

The classification accuracy employed refers to the proportion of correctly classified time series instances relative to the total number of instances in each experimental dataset. It directly reflects the capability of the chosen feature, SlpEn, to capture and represent the distinguishing characteristics of different classes in the time series data. A higher classification accuracy indicates that the model is more effective in correctly identifying the classes of time series instances.

(1) $$\mathrm{Accuracy}=\frac{\mathrm{Number}\;\mathrm{of}\;\mathrm{Correctly}\;\mathrm{Classified}\;\mathrm{Instances}}{\mathrm{Total}\;\mathrm{Number}\;\mathrm{of}\;\mathrm{Instances}}=\frac{TP+TN}{TP+TN+FP+FN}$$

where:

- TP (True Positives) are instances correctly identified as belonging to a particular class.
- TN (True Negatives) are instances correctly identified as not belonging to that class.
- FP (False Positives) are instances incorrectly identified as belonging to that class.
- FN (False Negatives) are instances incorrectly identified as not belonging to that class.

### 2.2. Slope Entropy

SlpEn [30] is an entropy calculation method based on extracting symbolic subsequences applying a set of thresholds to amplitude differences between consecutive time series samples. To the resulting histogram of relative frequencies of each subsequence, a Shannon entropy-like expression is then applied to acquire the final SlpEn result. This method is applied to an input time series x given input parameters *N*, *m*, γ and δ. Namely, the objective is to compute SlpEn(N,m,γ,δ), as described next.

We consider the input time series as an N − length vector x containing a set of samples $x_{i}$, defined as $x=\{x_{0},x_{1},x_{2},\ldots ,x_{N-1}\}$, $x_{i}\in R$, 0≤i<N. This time series is iteratively divided into overlapping data epochs of length *m*, $x_{j}=\left\{x_{j+0},x_{j+1},\ldots ,x_{j+m-1}\right\}$, 0≤j<N−m+1, *j* increased after each iteration as j→j+1.

Then, for each of these subsequences, an associated symbolic pattern is created, $x_{j}\to y_{j}$, with $y_{j}=\left\{y_{0}=f\left(x_{j+1}-x_{j+0}\right),y_{1}=f\left(x_{j+2}-x_{j+1}\right)\ldots ,y_{m-2}=f\left(x_{j+m-1}-x_{j+m-2}\right)\right\}$, using symbols from a set S={+2,+1,0,−1,−2} (or any other alphabet containing 5 different and unique symbols). These symbols are assigned by a thresholding function *f*, based on two thresholds γ and δ that in principle can take any positive real value, but with δ<γ, and the following rules (for 0≤k<m−1):

- If $x_{j+k+1}-x_{j+k}$ > γ, the symbol assigned to the current active symbolic pattern position is +2 (or just 2), $y_{k}=\left\{+2\right\}$.
- Else, if $x_{j+k+1}-x_{j+k}$ > δ, the symbol assigned to the current active symbolic pattern position is +1 (or just 1), $y_{k}=\left\{+1\right\}$.
- Else, if $|x_{j+k+1}-x_{j+k}|\leq \delta$, the case when two consecutive values are very similar (depending on threshold δ), which includes the case for ties when δ=0 [61], the symbol assigned to the current active symbolic pattern position is 0, $y_{k}=\left\{0\right\}$.
- Else, if $x_{j+k+1}-x_{j+k}<-\delta$ but $x_{j+k+1}-x_{j+k}\geq -\gamma$, the symbol assigned to the current active symbolic pattern position is −1, $y_{k}=\left\{-1\right\}$.
- Otherwise, the symbol assigned is −2, region where $x_{j+k+1}-x_{j+k}<-\gamma$, $y_{k}=\left\{-2\right\}$.

**Figure 1:**
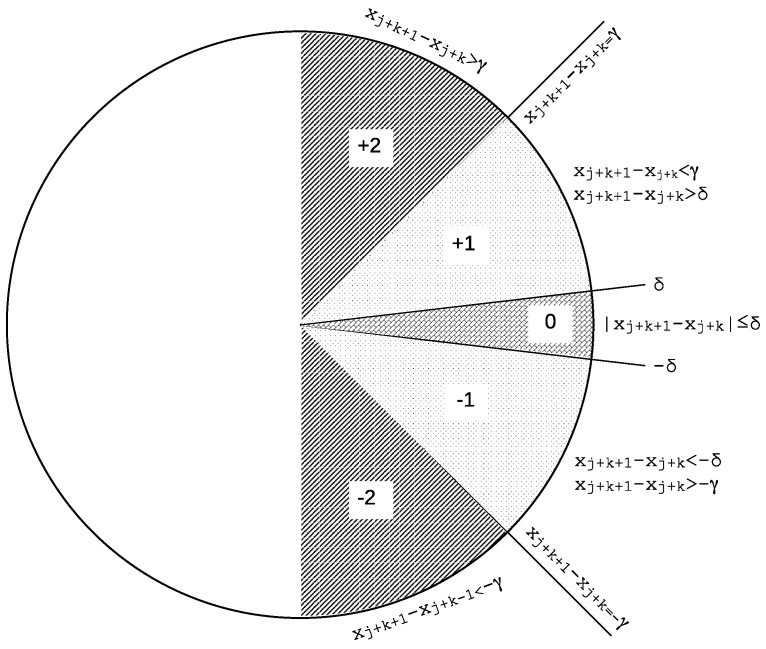
Graphical representation of the standard SlpEn thresholding process. This is a symmetric approach that uses the same absolute values for the thresholds in case of positive and negative gradients (an infinite gradient would correspond to a vertical line).

Graphically, the thresholding regions described above can be represented as shown in Figure 1. This is the standard symmetric SlpEn approach described in its original paper, and the one used so far in other scientific studies.

Once the computation of all the symbolic patterns has been completed, the total number of occurrences for each one is calculated (histogram bin height) and then normalised by the number of unique different patterns found. Each resulting value, for similarity with other methods, is referred to as $p_{k}$. Finally, the SlpEn for x using input parameters m,δ,γ, is obtained from a Shannon entropy expression:

(2) $$\mathrm{SlpEn}\left(x,m,\gamma ,\delta \right)=-\sum_{\forall k}p_{k}\log\;p_{k}$$

In principle, the role of threshold δ is to account for possible ties [30], and the role of threshold γ is to distinguish between high and low consecutive samples’ gradients. In its standard configuration described in the SlpEn original paper, δ was recommended to be a small value, 0.001, and γ, depending on input time series normalisation, to be a constant value in the vicinity of 1.0. Moreover, these thresholds were used for both positive and negative gradients, simply changing the sign. This is the symmetrical baseline scheme that has demonstrated its good performance in terms of classification accuracy in a number of studies already, like [9,33,62,63].

**Figure 2:**
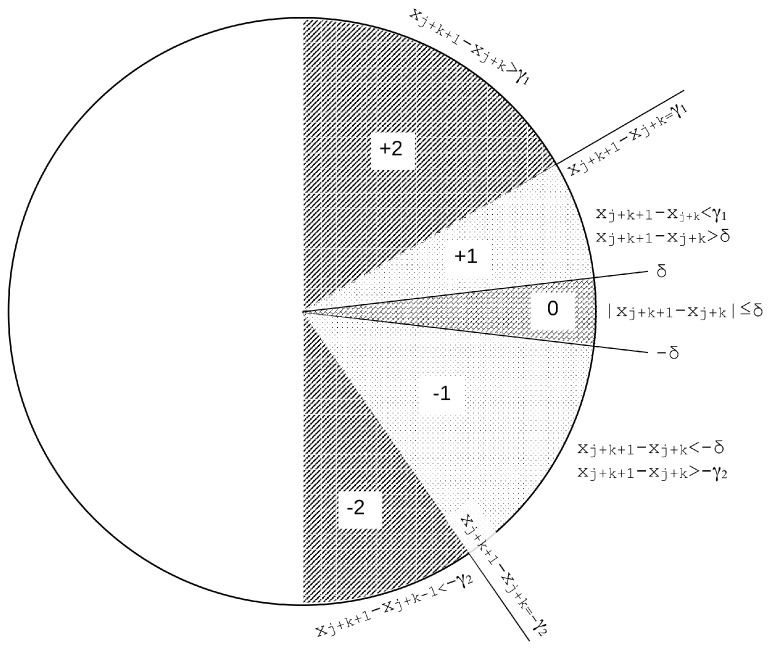
Graphical representation of the SlpEn thresholding process modification. This is an asymmetric approach that uses different absolute values for the thresholds in case of positive and negative gradients (no modifications for δ). If we wanted to remove δ from the method, that would just entail no {0} region, it would be absorbed by regions {+1} and {−1} with boundary at 0 slope line.

However, what remains to be studied is the impact on such performance that a finer tuning of the input parameters δ and γ could have, and that is what is investigated in the present paper. We propose to modify the SlpEn standard algorithm by considering different thresholds for positive and negative gradients and other variations like omitting the δ parameter, as described in the next Section 3. This is the possible SlpEn variation suggested to improve its classification potential. Graphically, this new approach is depicted in Figure 2 using two values for the γ parameter, $\gamma_{1}$ and $\gamma_{2}$.

Example: Let us take the same input time series used in [11], x={4,7,9,10,6,11,3}. If γ=2.5, δ=0.001, and embedded dimension *m* is set to 4, this results in 4 subsequences that can be extracted from x:

- $x_{0}=\left\{4,7,9,10\right\}$. In order to obtain the symbolic representation of this subsequence, we compute 7−4=3,9−7=2,10−9=1. Applying the thresholding method described above results in a symbolic pattern $y_{0}=\left\{+2,+1,+1\right\}$.
- $x_{1}=\left\{7,9,10,6\right\}$. In order to obtain the symbolic representation of this subsequence, we compute 9−7=2,10−9=1,6−10=−4. Applying the thresholding method described above results in a symbolic pattern $y_{1}=\left\{+1,+1,-2\right\}$.
- $x_{2}=\left\{9,10,6,11\right\}$. In order to obtain the symbolic representation of this subsequence, we compute 10−9=1,6−10=−4,11−6=5. Applying the thresholding method described above results in a symbolic pattern $y_{2}=\left\{+1,-2,+2\right\}$.
- $x_{3}=\left\{10,6,11,3\right\}$. In order to obtain the symbolic representation of this subsequence, we compute 6−10=−4,11−6=5,3−11=−8. Applying the thresholding method described above results in a symbolic pattern $y_{3}=\left\{-2,+2,-2\right\}$.

Since we have found 4 different patterns, the values for $p_{k}$ in Equation (2) are $p_{0}=p_{1}=p_{2}=p_{3}=\frac{1}{4}$. Therefore, the final SlpEn result is:

(3) $$\mathrm{SlpEn}\left(x,4,2.5,0.001\right)=-4*\frac{1}{4}\log\;\frac{1}{4}=1.3863$$

## 3. Experiments and Results

### 3.1. Experiments

In order to fully characterise the behaviour of SlpEn using an asymmetrical scheme for the input parameters, conducting a grid search for all possible input parameter values would be necessary. However, due to its substantial computational cost, such an exhaustive search was not feasible.

To strike a balance between the objectives of the paper and the temporal demands of the experiments, we opted to limit the scope. Accordingly, we maintained symmetry in the δ parameter and introduced a modification for the γ parameter. Specifically, we considered two values: $\gamma_{1}$ for positive gradients and $\gamma_{2}$ for negative gradients. The grid search for these parameters involved varying *m* between 3 and 9 (with a step of 1), δ between 0.05 and 1.00 (without exceeding $\gamma_{1}$ or $\gamma_{2}$), and $\gamma_{1}$ and $\gamma_{2}$ between 0.05 and 1.00 (in increments of 0.05). This grid search yielded the optimal input parameter configuration for achieving maximum classification accuracy when comparing the two classes in each complete dataset using SlpEn. On a Microsoft Windows 10 machine with an Intel Core i7 10th generation processor and 64 GB of RAM, utilising the Python programming language, the computation times for the asymmetric experiments were, for instance, 11,503 s for dataset Worms two-class, and 1342 s for the PAF prediction dataset.

To further mitigate computational costs, we adopted a strategy similar to the approach outlined in [34]. Specifically, we conducted additional experiments where the δ parameter was omitted, reducing the complexity of the grid search by one order of magnitude and the computational time accordingly.

We proceeded with a second set of experiments under the same conditions as stated above to evaluate performance in scenarios that align more closely with real-world classification applications. For this purpose, we allocated a random 30% of each dataset for training (used during the grid search to identify the optimal input parameters) and reserved the remaining 70% for validation. Each experiment was repeated ten times, and the reported results in the following section represent the average classification performance along with their corresponding standard deviations. This approach allowed us to assess the generalisation capabilities of the experiments while avoiding the pitfalls of parameter overfitting.

The performance of the methods tested was quantified in terms of classification accuracy: percentage of correctly classified time series based on a SlpEn feature for each experimental dataset [64].

### 3.2. Results

Table 1 and Table 2 present the classification accuracy achieved using the asymmetric SlpEn version detailed in the previous subsection. In Table 1, we employed a symmetric version of δ to mitigate computational costs while introducing two asymmetric versions of γ. Table 2 showcases results from an alternative approach where δ was completely omitted from the algorithm, following the methodology of [34]. These tables juxtapose our results with those obtained using the standard (baseline) SlpEn method, allowing for a visual assessment of the potential improvements in terms of time series classification accuracy. Additionally, both tables incorporate a column featuring the optimal parameter configuration determined through a comprehensive grid search.

Table 3 reports the results of the second set of experiments, utilising a training subset comprising a random 30% of the time series and a validation subset encompassing the remaining 70%. This approach was applied to both the cases with and without δ, each repeated across 10 random realisations for every dataset.

## 4. Discussion

The original SlpEn method [30] proposed a symmetrical scheme for the input thresholding parameter γ, and also for δ, but this last one keeping its value constant at 0.001 or −0.001. The intention with δ was, in principle, to simply account for similar consecutive sample values that were hypothesised to create ambiguities when computing symbolic representations [65]. However, later studies demonstrated that ties do not exert a significant negative impact on classification accuracy [61], and therefore, we subsequently studied if, as a first step for SlpEn simplification, that parameter could just be omitted [34]. Obviously, removing a parameter will never improve classification performance if the tests are carried out under the same conditions; at most, it can equal the accuracy achieved (removing a parameter is equivalent to including its 0 value in a grid search). What is crucial is to assess if there is a significant performance degradation and whether the saved computational cost pays off.

This work is a continuation of that first SlpEn characterisation study to better understand how SlpEn can be improved both in terms of computational burden and classification accuracy. In this regard, the results presented in Table 1, Table 2 and Table 3 offer valuable insights into the performance of the asymmetrical SlpEn variant compared to the baseline method. The two modifications of the asymmetrical approach, with and without the δ parameter, for complete datasets or for training–validation partitions, were systematically examined across multiple datasets.

From Table 1, it is evident that introducing asymmetry in the γ parameter while maintaining symmetry in δ (albeit at different values) led to improved classification accuracy across several datasets, as could be expected. Notably, the accuracy enhancements achieved by this approach were consistent across all datasets tested, reinforcing the validity of this approach in terms of classification accuracy. These improvements span from just 1% for the Bern–Barcelona database to a very significant 22% for the Worms two–class dataset.

Table 2 introduces a different perspective. By entirely omitting the δ parameter, the complexity of the grid search was significantly reduced. In this case, the approach managed to maintain or even exceed the accuracy levels attained by the baseline method across the experimental datasets, although, as expected, the accuracy was lower than when using δ (both cases in Table 1). This result aligns with the findings in [34], underscoring the feasibility of adopting this strategy to reduce computational costs, knowing the accuracy will not be severely affected.

Table 3 encapsulates the mean and standard deviation classification accuracy resulting from the training–validation experiments. In general, the accuracy achieved in these experiments is lower than in Table 1 and Table 2 since the optimal parameters are computed using only 30% of the datasets. However, the observed consistency in performance supports the generalisation capabilities of both asymmetrical SlpEn approaches, corroborating their effectiveness in real-world classification tasks. There is a specific case, the Ford A dataset, for which the results seem to be counterintuitive; they improved when removing the δ parameter. However, this is completely normal since the training and test datasets were randomly generated 10 times for each experiment, and therefore, they were supposed not to contain the same records. Thus, by coincidence, the actual partial datasets could have been more favourable for classification in one case than in the other. If the performance is additionally very similar with and without δ; there can be a kind of inversion effect as in this case.

Overall, the outcomes of the experiments highlight the potential benefits of asymmetrical parameter configurations in the SlpEn method. The observed accuracy improvements underscore the importance of tailoring parameter settings to the dataset characteristics, enabling better exploitation of underlying patterns. However, the main weakness of the variations proposed is the significant increment in computational cost. Although the computations using the standard SlpEn method took a few seconds or minutes at most, adding the asymmetric version implied hours of computational time. We recommend sticking to the standard SlpEn method to achieve a very good trade-off between time and accuracy, and only in specific cases where accuracy is of utmost importance or where the basic method is unable to yield a satisfying classification, resorting to asymmetric parameter versions.

## 5. Conclusions

This study has addressed an exploration of an asymmetrical variant of the SlpEn method aimed at enhancing time series classification accuracy. Our experimentation utilised two distinctive approaches: one retaining symmetry exclusively in the δ parameter while introducing asymmetry solely in the γ parameter, and the other adopting the methodology outlined in [34], omitting the δ parameter entirely but introducing asymmetry in γ. The study also includes a training–validation subset scheme. The findings offer valuable insights into the potential advantages and trade-offs associated with these asymmetrical SlpEn variants.

In general, the results emphasise that a higher degree of parameter customisation consistently leads to improved classification accuracy across all cases. This level of flexibility enables enhanced pattern recognition and classification performance. Researchers are encouraged to consider asymmetrical configurations carefully, tailoring the SlpEn method to match their specific requirements, accounting for dataset characteristics and computational constraints. Notably, when the baseline SlpEn method already achieves high classification accuracy, further improvements via parameter expansion may be limited.

Additionally, our findings suggest a hypothesis worth exploring further: Narrowband signals, such as those from the Fantasia, Ford A, PAF, and Worms datasets, exhibit a more substantial potential for increased classification accuracy compared to broadband signals like EEG data. Future studies should delve deeper into this hypothesis to gain a more comprehensive understanding.

Regarding computational cost optimisations, it is evident that potential enhancements may involve refining the SlpEn calculation algorithm, employing faster programming languages, utilising more powerful computing hardware, or exploring parallel processing techniques. Furthermore, efficient grid search strategies beyond brute-force methods or entirely novel input parameter optimisation schemes could significantly contribute to the field. Future research endeavours are encouraged to investigate these optimisation avenues for the proposed methods, extending their applicability to diverse domains and datasets. Such efforts will surely contribute to advancing the characterisation of SlpEn-based time series classification methods.

## Figures and Tables

**Table 1:** Classification accuracy achieved of the asymmetrical γ with symmetric δ SlpEn version, for each dataset. Baseline results are also reported for comparison, as well as the optimal input parameter configuration that maximises accuracy.

Dataset	Baseline	SlpEn with δ	Parameters
Bern–Barcelona	80%	81%	m=7, $\gamma_{1}=0.35$, $\gamma_{2}=-0.20$, δ=0.10
Fantasia	85%	89%	m=5, $\gamma_{1}=0.50$, $\gamma_{2}=-0.45$, δ=0.05
Ford A	83%	94%	m=3, $\gamma_{1}=0.45$, $\gamma_{2}=-0.20$, δ=0.05
House Twenty	95%	97%	m=5, $\gamma_{1}=0.20$, $\gamma_{2}=-0.20$, δ=0.50
PAF prediction	76%	84%	m=6, $\gamma_{1}=0.55$, $\gamma_{2}=-0.20$, δ=0.10
Worms two-class	70%	92%	m=3, $\gamma_{1}=0.80$, $\gamma_{2}=-0.80$, δ=0.75
Bonn EEG	94%	95%	m=9, $\gamma_{1}=0.20$, $\gamma_{2}=-0.85$, δ=0.05
Synth1	92%	95%	m=8, $\gamma_{1}=0.70$, $\gamma_{2}=-0.75$, δ=0.05
Synth2	87%	89%	m=8, $\gamma_{1}=0.75$, $\gamma_{2}=-0.70$, δ=0.05
Synth3	95%	95%	m=8, $\gamma_{1}=0.60$, $\gamma_{2}=-0.70$, δ=0.05

**Table 2:** Classification accuracy achieved of the asymmetrical γ without δ SlpEn version, for each dataset. Baseline results are also reported for comparison, as well as the optimal input parameter configuration that maximises accuracy.

Dataset	Baseline	SlpEn without δ	Parameters
Bern–Barcelona	76%	77%	m=7, $\gamma_{1}=0.20$, $\gamma_{2}=-0.20$
Fantasia	82%	89%	m=5, $\gamma_{1}=0.50$, $\gamma_{2}=-0.45$
Ford A	82%	94%	m=3, $\gamma_{1}=0.20$, $\gamma_{2}=-0.45$
House Twenty	95%	95%	m=7, $\gamma_{1}=0.60$, $\gamma_{2}=-0.65$
PAF prediction	76%	80%	m=6, $\gamma_{1}=0.55$, $\gamma_{2}=-0.20$
Worms two-class	69%	72%	m=6, $\gamma_{1}=0.30$, $\gamma_{2}=-0.45$
Bonn EEG	93%	93%	m=9, $\gamma_{1}=0.20$, $\gamma_{2}=-0.80$
Synth1	89%	89%	m=8, $\gamma_{1}=0.65$, $\gamma_{2}=-0.70$
Synth2	87%	89%	m=8, $\gamma_{1}=0.75$, $\gamma_{2}=-0.70$
Synth3	86%	89%	m=8, $\gamma_{1}=0.65$, $\gamma_{2}=-0.70$

**Table 3:** Mean and standard deviation of classification accuracy using the asymmetrical approach with and without δ after repeating the training–testing experiment 10 times.

Dataset	SlpEn with δ		SlpEn without δ	
	Mean	sd	Mea	sd
Bern–Barcelona	76.81%	1.82	68.04%	2.98
Fantasia	78.15%	3.66	75.18%	3.63
Ford A	93.87%	2.30	93.98%	2.00
House Twenty	97.36%	0.00	75.25%	1.44
PAF prediction	72.36%	2.77	65.62%	2.17
Worms two-class	92.02%	1.18	69.49%	1.63
Bonn EEG	85.41%	4.42	81.63%	7.01
Synth1	75.70%	3.21	69.54%	5.26
Synth2	71.02%	2.11	68.34%	4.80
Synth3	72.06%	3.31	70.63%	3.60

## Data Availability

All real datasets used in this paper are well known and publicly available.
